# Anti-cholinesterase, anti-inflammatory and antioxidant properties of *Combretum micranthum* G. Don: Potential implications in neurodegenerative disease

**DOI:** 10.1016/j.ibneur.2022.12.001

**Published:** 2022-12-08

**Authors:** Mabozou Kpemissi, Yendube T. Kantati, Veeresh Prabhakar Veerapur, Kwashie Eklu-Gadegbeku, Zurina Hassan

**Affiliations:** a Centre for Drug Research, Universiti Sains Malaysia, 11800 Minden, Penang, Malaysia; b University of Lomé, Togo; c Sree Siddaganga College of Pharmacy, B.H. Road, Tumkur 572 102, Karnataka, India

**Keywords:** *Combretum micranthum*, Anti-inflammatory, Antioxidant, Anticholinesterase, Neuroprotection

## Abstract

**Background:**

Brain damage is a severe and common pathology that leads to life-threatening diseases. Despite development in the research, the medical evidence of the effectiveness of potential neuroprotective medicines is insufficient. As a result, there is an immense and urgent demand for promising medication. For millennia, herbal remedies were a fundamental aspect of medical treatments. *Combretum micranthum* (CM), a plant of the family Combretaceae in sub-Saharan Africa, has been utilized in folklore medicine to cure diverse human ailments. In order to develop a neuroprotective phytomedicine, the current research was undertaken to explore the antioxidant, anti-inflammatory, anticholinesterase and neuroprotective potential of CM extract*.*

**Methods:**

Colorimetric methods were used to determine CM antioxidant activity, in-vitro protein denaturation and membrane destabilization assays were used to evaluate its anti-inflammatory capacity, anticholinesterase activity was carried out using Ellman’s method, and neuroprotective potential was assessed on brain homogenate stressed with ferric chloride and ascorbic acid (FeCl_2_-AA) by assessing the lipoperoxidation biomarker malondialdehyde (MDA).

**Results:**

In Ferric Reducing Antioxidant Power (IC_50_ = 27.15 ± 0.06 µg/mL) and Total Antioxidant Capacity (IC_50_ = 31.13 ± 0.02 µg/mL), CM extract demonstrated strong antioxidant activity. Anti-inflammatory effect were improved in heat-induced Egg albumin and BSA denaturation (IC _50_ = 46.35 ± 1.53 and 23.94 ± 1.10 µg/mL) as well as heat and hypotonia induced membrane destabilization (IC _50_ = 20.96 ± 0.11 and 16.75 ± 0.94 µg/mL).

CM extract showed strong anticholinesterase activity (IC _50_ = 59.85 ± 0.91 µg/mL). In an ex-vivo neuroprotective model, CM extract showed substantial inhibition (p < 0.001) of oxidative damage caused by FeCl_2_-AA in brain tissue.

**Conclusion:**

*C. micranthum* may be a good candidate for its probable neuroprotective potential. Its neuroprotective benefits might be attributed to its antioxidant, anti-inflammatory and anticholinesterase effects.

## 1 Introduction

In recent decades, life expectancy increased, but this progress has not been expressed in well-being. Neurodegenerative disease (ND) include Alzheimer's disease, Huntington's disease, Parkinson's disease, and amyotrophic lateral sclerosis are, without a doubt, the most severe health issues that face the elderly (Welcome, 2020, Karaboga and Sezginturk, 2022). Every 3 s, someone develops dementia (Karaboga and Sezginturk, 2022). In 2020, fifty million or more of the world's population were living with ND and this number is projected to reach 100 million by 2040 (Karaboga and Sezginturk, 2022). Managing the current demands for ND treatment poses an enormous challenge to medical systems globally (Tiang et al. 2020). Furthermore, another major healthcare concern in the twenty-first decade is the global incidence of ND and accompanying memory deficits. A deﬁnitive treatment to prevent the incidence of ND is still to be discovered (Ilesanmi et al. 2022). In spite of significant advances in knowing the processes behind ND, therapy is still unsuccessful (Subedi and Gaire, 2021). There's really still an attempt to identify safe drugs to treat ND (Onyebuchi et al., 2022). Herbal medicines can play an essential role through drug development, and their research is a rational search strategy for novel medications (Junchi et al., 2022, Welcome, 2020). Herbs are a rich source of active natural chemicals, and many of them are beneficial and safe for ND prevention (Orhan et al., 2021, Chauhan et al., 2022). *Combretum micranthum* (CM) commonly called Kinkeliba is an African medicinal plant with several ethnopharmacological activities (Kpemissi et al., 2019b). Our previous research work highlights this plant as a potential source of renoprotective compounds in different nephrotoxicity models (Kpemissi et al., 2022, Kpemissi et al., 2019a; Kpemissi et al., 2020b, Kpemissi et al., 2020a). Phytochemical and in silico screening revealed that CM extract contains flavonoids that inhibit nuclear factor kappa B and soluble epoxide hydrolase (Kpemissi et al., 2019a) while stimulating peroxisome proliferator-activated receptors alpha and gamma (Kpemissi et al., 2020b), which are potential targets in neuroprotection (Certo et al., 2015, Wang et al., 2018, Xie et al., 2020). Furthermore, flavonoid compounds appear to have therapeutic targets which are potential in neuroprotection (Saraswat et al., 2020, Welcome, 2020). Recently, an ethnobotanical survey reported that CM is widely applied in brain disorders such as dementia and schizophrenia (Kantati et al., 2016, Mounkoro et al., 2020). There is no scientific evidence to prove this matter. Given above findings, we thought it would be worthwhile to carry a complete investigation combining drug development methodologies as well as in vitro and ex vivo tests to establish the potential of CM in the treatment of neurodegenerative illnesses.The current work aims to investigate anti-inflammatory, antioxidant and anticholinesterase properties of CM in order to enhance its use in ND with plant-based drugs.

## 2 Material and methods

### 2.1 Chemicals

Malondialdehyde (MDA), ascorbic acid, methyl-2-phenylindole, nitric oxide, Ferric chloride, 2,6-di-tert-butyl 4-methylphenol, methanol, 5,5-dithiobis-2-nitrobenzoic acid, Bradford reagent, dimethyl sulphoxide (DMSO), acethylthiocholine iodide (ATCI), butyrylthiocholine iodide (BTCI), acetylcholinesterase (AChE), butyrylcholinesterase (BuChE), and bovine serum albumin (BSA) were procured from Sigma-Aldrich. All other chemicals and reagents were of analytical grade.

### 2.2 Plant materials and extraction

Professor Koffi AKPAGANA of the University of Lome's Botany Department identified *C. micranthum* leaves were harvested in the Alibi I zone of Tchamba's northwestern region. A voucher specimen (N° TOGO151088) was kept in the herbarium of the Institute. The extraction was done as before, with a yield of 12.15% w/w (Kpemissi et al., 2019a, Kpemissi et al., 2019b, Kpemissi et al., 2020b, Kpemissi et al., 2020a).

### 2.3 Experimental animals

Adult male albino rats of the Wistar strain, weighing between 200 and 250 g were used. They were housed in a controlled environment with a 12 h light: dark cycle and were given access to standard laboratory food and water ad libitum. All experiments involving animals were conducted in strict compliance with ethical committee of the University of Lome (N° SBM/UL/20/RF2135).

### 2.4 In-vitro anti-inﬂammatory activities

#### 2.4.1 Protein denaturation inhibition assay

##### 2.4.1.1 BSA anti denaturation assay

The method of Saleem et al. (2020) was used. The reaction mixture was composed of 450 μL of 5% w/v BSA solution and 50 μL of CM extract or standard drug solutions (aspirin and diclofenac sodium) at different concentrations (25–400 µg/mL). The sample mixture was kept for 15 min at 37 °C and then for 5 min at 70 °C. After cooling he samples, 2.5 mL PBS (pH 6.3) was added to the mixture. The absorbance of the test and control samples were measured at 660 nm. The experiment was run three times and the % inhibition in protein denaturation was calculated using the equation

$$\mathrm{Percentage inhibition}=\frac{K_{0}-K_{t}}{K_{0}}\times 100$$

Where, K_0_ = absorbance value of control, K_t_ = absorbance value of CM extract or standards.

The IC_50_ values of the CM extract, aspirin, and diclofenac sodium were generated in GraphPad Prism-9 (San Diego, CA, USA) using Fit spline analysis.

##### 2.4.1.2 Egg albumin (EA) denaturation inhibition assay

This assay has been done as described previously (Dadoriya et al. 2020). The reaction mixture was composed of 0.2 mL of fresh hen’s egg albumin, 2.8 mL phosphate buffer saline (pH 6.4) and 2 mL of various concentrations (25–400 µg/mL) of CM extract or standard drugs. All the samples were set aside at 37 °C for 25 min followed by heating for 5 min at 70 °C. The cooled solutions were centrifuged at 3000 rpm for 10 min. Then the absorbance of supernatant solutions was measured at 660 nm. The % of inhibition and IC_50_ values were calculated as mentioned in BSA denaturation inhibition assay procedure.

#### 2.4.2 Red blood cell (RBC) membrane stabilization assay

The anti-inflammatory activity was assessed using RBC membrane stability. The equal volume of rat blood and 0.9% w/v normal saline was taken in heparinized tubes. The blood was centrifugated for 10 min at 3000 rpm. Then the suspended cells were washed carefully with saline three times. Finally, 10% v/v cell suspension was prepared using saline (Joshi et al., 2020) and used in hypotonicity- and heat-induced hemolysis assay.

##### 2.4.2.1 Hypotonicity-induced hemolysis

This experiment was executed using the method as described in the literature (Saleem et al., 2020). The investigational mixture contained 0.5 mL of CM extract or standard drug solutions (25–400 µg/mL), 1 mL of 150 mM phosphate buffer of pH 7.4, 2 mL of 0.36% w/v of hyposaline, and 0.5 mL RBC cell suspension. The sample mixtures were kept aside for 30 min at 37 °C, then cooled and centrifugated for 10 min at 3000 rpm. The absorbance was measured at 560 nm using UV spectrophotometer. The % inhibition of hemolysis and IC_50_ values were calculated as mentioned in above procedure.

##### 2.4.2.2 Heat-induced hemolysis

The assay mixture contained 1 mL of RBC suspension and 1 mL CM extract or standard drug solutions (25–400 µg/mL). The tube samples were kepet at 70 ˚C in the bath for 30 min. After cooling and centrifuging for 5 min at 3000 rpm, the absorbance was measured at 560 nm using UV spectrophotometer (Dadoriya et al., 2020). In addition, the % inhibition of hemolysis and IC_50_ values were calculated.

### 2.5 *In-vitro* steady-state antioxidant activities

#### 2.5.1 Ferric reducing antioxidant power (FRAP) assay

The power of the extract to reduce ferric ions was analyzed using the reported method (Zhang et al., 2020, Annadurai et al., 2021). Acetate buffer of pH 3.6 (300 mM), tripyridyl triazine solution (10.0 mM) and ferric chloride solution (20 mM) were mixed in a 10:1:1 vol ratio to make the FRAP reagent. The freshly prepared FRAP reagent (2 mL) was combined with various amounts (10–500 µg/mL) of plant extract or standards. The samples were kepet in dark place for 30 min at 37 °C and The absorbance was measured at 593 nm. Fresh working solutions of ferrous sulfate was used for the calibration purpose. From the linear calibration curve, ability of samples to reduce ferric ions was mesured. The EC_50_ values of the CM extract, ascorbic acid and quercetin were calculated using GraphPad Prism-9 software using Fit spline analysis.

#### 2.5.2 Total antioxidant capacity assay (TAC)

TAC assay was carried out according the reported method (Annadurai et al., 2021). The assay mixture contained 300 μL of CM extract and 3000 μL of phosphomolybdate reagent were kept at 95 °C for one and half hours. After centrifugation at 3000 rpm for 5 min and absorbance of the cool supernatant solution was recorded at 765 nm. The TAC of the CM extract and tested standards were reported as the EC_50_ values.

### 2.6 *Ex-vivo* antioxidant and neuroprotective studies

#### 2.6.1 Ascorbic acid/Fe ^2+^ (FeCl_2_-AA) induced lipid peroxidation in rat brain homogenate

The anti-lipid peroxidation effect of CM extract was studied following the reported method (Kpemissi et al., 2015, Kpemissi et al., 2019b). Rats were killed by decapitation and their brain tissues were quickly removed. A 4 g portion of brain tissue was sliced and then homogenized with 20 mL of hydrochloric acid-potassium chloride buffer (150 mM), pH 7.4. The investigational tubes contained 500 μL of brain homogenate, 200 μL of buffer (pH 7.4), 100 μL of 0.1 mM ascorbic acid, 100 μL of 4 mM FeCl_2_ and 100 μL of different amount of CM extract or quercetin. The samples were kept at 37 °C for one hour. After the incubation, the malondialdehyde (MDA) concentration in the samples was estimated as previously described using 1,1,3,3-tetra-methoxypropane to make a standard curve (Kpemissi et al., 2019b).

### 2.7 *In vitro* anticholinesterase assay

The CM extract was evaluated for its potential in vitro anticholinesterase activity using Ellman's method (Orhan et al. 2021). The standard was iso-OMPA. The plant extract was prepared in 100% DMSO. In a 96-well microplate, 140 μL of 0.1 M PBS (pH 7.5), 20 μL of samples, and a 0.09 unit/mL AChE or BuChE were added. Samples were kept at 25 °C for 15 min, 10 μL of DTNB (10 mM) and ATCI or BTCI (14 mM) were added. The plate was stirred for 10 s before taking absorbance measurements at 412 nm for 30 min using a microplate spectrophotometer (Thermo Scientific, USA). The formula used to obtain the inhibition percentage was:

$\mathrm{Percentage inhibition}=\frac{K_{0}-K_{t}}{K_{0}}\times 100$

With K_0_ = absorbance of control, K_t_ = absorbance of CM extract or standards.

The half maximal inhibitory concentration of CM extract, aspirin, and diclofenac sodium were generated in GraphPad Prism-8 using Fit spline analysis.

### 2.8 Total protein estimation in brain tissue

Bradford's technique was used to find the quantity of protein within samples (Kpemissi et al., 2019b).

### 2.9 Statistical study

All data are treat in Graph Pad Prism 9 software and presented as means ± SEM. One-way analysis of variance followed by Tukey's test was used. Probability values p ≤ 0.05 were considered statistically signiﬁcant.

## 3 Results

### 3.1 In-vitro anti-inﬂammatory activities

#### 3.1.1 Protein anti-denaturation assay

The IC_50_ values of CM extract and standards (aspirin and diclofenac sodium) were reported in Table 1. These IC_50_ values indicated their anti-inflammatory power in heat-induced protein denaturation assay protocols. CM extract exhibited almost nearer IC_50_ values than that of standard tested drugs.

**Table 1:** Anti-inflammatory effect of *C. micranthum* extract in denaturation of egg albumin (EA) and bovine serum albumin (BSA) inhibition assay.

**Substances**	**IC**_**50**_**(µg/mL)**	
	**EA**	**BSA**
***C. micranthum***	46.35 ± 1.53	23.94 ± 1.10
**Aspirin**	39.92 ± 1.63	14.37 ± 0.64
**Diclofenac sodium**	42.05 ± 0.94	13.71 ± 0.08

All the values expressed in Mean ± SEM.

#### 3.1.2 Red blood cell (RBC) membrane stabilization assay

CM extract exhibited good RBC membrane stabilizing properties as evident by lower IC_50_ values as expressed in Table 2. The observed membrane stabilization potential by the CM extract was found to be comparable with known anti-inflammatory drugs in hypotonia- and heat-induced hemolysis models.

**Table 2:** Anti-inflammatory effect of *C. micranthum* extract in membrane stabilization of red blood cells.

**Substances**	**IC**_**50**_**(µg/mL)**	
	**Hypotonia**	**Heat**
***C. micranthum***	16.75 ± 0.94	20.96 ± 0.11
**Aspirin**	16.52 ± 0.42	18.28 ± 0.25
**Diclofenac sodium**	17.68 ± 0,96	17.33 ± 0.22

Results expressed as Means ± SEM, (n = 3 replicates)

### 3.2 In-vitro steady-state antioxidant activities

#### 3.2.1 Ferric reducing antioxidant power (FRAP) assay

The reduction of the ferric tripyridyltriazine complex (Fe^3+^-TPTZ) to Fe^2+^-TPTZ complex by CM extract and the standard compounds was tabulated in Table 3. The percentages reduction by CM extract and stantards (quercetin and ascorbic acid) were found to be 97.59 ± 0.17%, 98.26 ± 0.73% and 75.24 ± 0.08% consecutively. The EC_50_ values of the CM extract (27.15 ± 0.06 µg/mL) was found to be better than quercetin (37.50 ± 0.28 µg/mL) and nearly similar to ascorbic acid (23.62 ± 0.02 µg/mL).

**Table 3:** Antioxidant activity of *C. micranthum* against TAC and FRAP radicals.

**Substances**	**EC**_**50**_**(µg/mL)**	
	**TAC**	**FRAP**
***C. micranthum***	31.13 ± 0.02	27.15 ± 0.06
**Quercetin**	30.11 ± 0.14	37.50 ± 0.28
**Ascorbic acid**	29.73 ± 0.05	23.62 ± 0.02

All the values expressed in Mean ± SEM.

#### 3.2.2 Total antioxidant capacity (TAC)

TAC of the CM extract determined was tabulated in Table 3. The CM extract showed a comparable TAC as evident by its EC_50_ (31.13 ± 0.02 µg/mL) when compared to quercetin (30.11 ± 0.14 µg/mL) and ascorbic acid (29.73 ± 0.05 µg/mL).

### 3.3 Ex-vivo antioxidant and organo-protection studies

#### 3.3.1 FeCl_2_-AA induced lipid peroxidation

Incubating brain tissue with FeCl_2_-ascorbic acid increased lipoperoxidation leading to MDA generation significantly (p 0.001). However, CM extract and quercetin significantly (p < 0.001) inhibited the lipid peroxidation in tested homogenate induced by FeCl_2_-AA (Table 4).

**Table 4:** Effect of *C. micranthum* extract on FeCl_2_-AA-induced lipoperoxidation on brain homogenate.

**Groups**	**Concentration (µg/mL)**	**MDA (nM/mg protein)**
**Normal control**		528.705 ± 0.005
**FeCl**_**2**_**-AA**		895.470 ± 0.050^###^
**FeCl**_**2**_**-AA** **+** ***C. micranthum***	25	536.285 ± 0.375***
	50	582.490 ± 0.039***
	100	782.570 ± 0.089***
	200	769.420 ± 1.360***
	400	745.905 ± 1.115***
**FeCl**_**2**_**-AA** **+** **Quercetin**	25	841.685 ± 1.825^¤¤¤^
	50	752.955 ± 0.715^¤¤¤^
	100	765.205 ± 1.825^¤¤¤^
	200	790.820 ± 1.970^¤¤¤^
	400	728.490 ± 0.040^¤¤¤^

The results are expressed as Means ± SEM (n = 3 replicates). ANOVA followed by Tukey's multiple comparison test. ### P < 0.001: FeCl2-AA vs Control, *** P < 0.001: FeCl2-AA vs CM; ¤ P < 0.05; ¤¤ P < 0.01; ¤¤¤ P < 0.001: FeCl2-AA vs Quercetin

#### 3.3.2 In vitro anticholinesterase activity of CM extract

The in vitro cholinesterase activity assay revealed CM extract has potential anticholinesterase action with IC_50_ values varied from 13.19 ± 1.20 µg/mL and 59.85 ± 0.91 µg/mL for AChE and BuChE activities, respectively. Table 5 shows percentage of inhibitions and IC_50_ of CM extract gains AChE and BuChE.

**Table 5:** *C. micranthum* extract and Iso-OMPA's percentage inhibition and IC_50_ against AChE and BuChE.

		**AChE inhibition activity**		**BuChE inhibition activity**	
**Substances**	**Concentration** **(µg/mL)**	**Inhibition** **(%)**	**IC**_**50**_ **(µg/mL)**	**Inhibition** **(%)**	**IC**_**50**_ **(µg/mL)**
	12.5	81.83 ± 1.70		23.56 ± 0.69	
***C. micranthum***	25	75.16 ± 1.28		56.28 ± 0.45	
	50	80.63 ± 0.73	**13.19 ± 1.20**	37.57 ± 1.70	**59.85 ± 0.91**
	100	87.25 ± 1.07		44.15 ± 0.76	
	200	92.93 ± 1.68		48.88 ± 1.04	
	500	98.65 ± 0.52		64.32 ± 1.21	
	12.5	81.83 ± 1.70		33.18 ± 1.97	
**Iso-OMPA**	25	75.16 ± 1.28		41.26 ± 0.49	
	50	80.63 ± 0.73		52.42 ± 1.20	
	100	87.25 ± 1.07	**12.58 ± 0.92**	71.65 ± 1.80	**41.00 ± 1.22**
	200	92.93 ± 1.68		89.14 ± 0.97	
	500	98.65 ± 0.52		97.01 ± 0.54	

The results are expressed as Means ± SEM (n = 3 replicates).

## 4 Discussion

Several pathogenic hypotheses have been proposed for ND, including the cholinergic, amyloid cascade, oxidative stress, neuroinflammatory, and tau protein (Tang et al., 2022; Onyebuchi et al., 2022). In this study, we aimed to evaluate the potential of CM on three ND hypotheses by investigating its anti-inflammatory, antioxidant, and anticholinesterase activities. The vast majority of ND are occasioned by oxidative stress induced by the overproduction of free radicals and stimulation of the inflammatory process (Ansari et al., 2020, Wu et al., 2020). Oxidative stress and inflammation are at the center of most chronic diseases and are the driving force behind ND processes (Wu et al., 2020, Tang et al., 2022). There is a strong link between anti-inflammatory, antioxidants and neuroprotection (Ojeaburu and Oriakhi, 2021, Tang et al., 2022). Because of the increasing demand for novel unique compounds, the neuroprotective actions of compounds identified from medicinal plants are an exciting step forward in the quest for effective protectors (Taqui et al., 2022). Finding molecules with antioxidant, anti-inflammatory and anticholinesterase properties is one of the most important goals in therapeutic research (Onyebuchi et al., 2022). *In vitro* testing is widely used as a preliminary step before performing in vivo confirmation. As of now, there are several in vitro approaches for assessing the antioxidant (Zhou et al., 2021) and anti-inflammatory potential (Protein denaturation and membrane stabilization assay) of plant-based products (Saleem et al., 2020). The antioxidant and anti-inflammatory potential of CM extract was evaluated by spectrophotometry using these in vitro models. During injury and pathogenic attack, the inflammatory response is a challenging state strongly related to discomfort and involves processes such as increased protein denaturation and cellular membrane rupture (Dadoriya et al., 2020). Further, tissue inflammation manifests the body's reaction to stress (Yuan et al., 2020). Once tissue cells are damaged, signaling molecules such as kinins, prostaglandins and histamine are released, thus mobilizing many of the body's defense cells (Saleem et al., 2020). Multiple experiments involving the inflammatory process are performed to test the effectiveness of medicines. Well known, protein denaturation is linked to inflammatory processes (Dadoriya et al., 2020). Denaturation of proteins is characterized by the loss of their tertiary and secondary structure. It is caused by a stressor like a strong base or acid, heat, an organic solvent, or a concentrated inorganic salt (Kar et al., 2012). Biological proteins lose their functionality when they are denatured (Joshi et al., 2020). As a result, any agent that can prevent protein denaturation could significantly reduce inflammation (Mouffouk et al., 2020). Several anti-inflammatory drugs, in varying degrees, suppress heat protein denaturation. The ability of CM extract to suppress protein denaturation was investigated to learn more about how it exerts its anti-inflammatory effect. In the current study, the CM extract considerably reduced protein denaturation (BSA and egg albumin), comparable to conventional treatments' results. The anti-inflammatory mechanism of action of CM extract via prevention of proteins denaturation could be explained based on previous findings. Indeed, Protein denaturation occurs during tissue injury or inflammatory process (Das et al., 2022). Numerous anti-inflammatory drugs deter thermally prompted protein denaturation (Anokwah et al., 2022). Protein denaturation is the principal cause of inflammatory and arthritic disorders that proceed to auto-antigens generation, which can initiate the auto-immune reaction that can converge onto the rheumatic arthritis (Das et al., 2022). Auto-antigens formation due to thermally induced protein denaturation causes the alterations in disulfide, hydrogen and electrostatic hydrophobic bonding (Anokwah et al., 2022). Williams et al. (2008), Elisha et al. (2016) have reported that BSA expresses antigen associated Type III hypersensitivity reactions which are associated to the many disorders. Thus, protection against protein denaturation, which was the main mechanism of action of non-steroidal anti-inflammatory drugs (NSAIDs) postulated by Mizushima (1964) before the discovery of their inhibitory effect on cyclooxygenase (Vane, 1971), may play an important role in the antirheumatic activity of NSAIDs (Kdpp et al., 2018). The ability of CM to inhibit thermal and hypotonic protein denaturation may contribute to its anti-inflammatory properties. Erythrocytes are a good model for assessing the anti-inflammatory properties of drugs in vitro (Saleem et al., 2020). The capacity of the extract to stabilize the erythrocyte membrane can be extended to the lysosomal membrane in vivo since the RBC membrane mimics the lysosomal membrane (Dadoriya et al., 2020). In order to decrease the inflammatory response, the lysosome membrane must be protected. It limits the release of lysosome components such as enzymes that boost inflammatory responses and cause tissue damage (Parameswari et al., 2019, Saleem et al., 2020). Thus, stabilizing the lysosome membrane would limit inflammatory processes by preventing the release of lysosomal mediators from active neutrophils (Anosike et al., 2019). Therefore stabilization of the lysosome membrane would prevent the release of inflammatory intermediates, thereby reducing cell stress and tissue damage (Umukoro et al., 2017). Several anti-inflammatory medications work by stabilizing the membrane of the lysosome (Anosike et al., 2019). In this study, CM extract inhibited the heat or hypotonicity-induced RBC hemolysis. Overall, our findings indicated that CM extract has potent anti-inflammatory effects.

The TAC of the CM extract was measured using the phosphomolybdate assay. In the presence of an antioxidant, molybdenum (VI) is reduced to molybdenum (V), generating a green phosphomolybdate (V) complex. (Chohra et al., 2020). This action has been observed in several plant components, including phenols and flavonoids. The FRAP assay highlights reducing power of antioxidants in a redox-related colorimetric method (Chohra et al., 2020). The FRAP test can therefore be used to determine compounds' reducing power and antioxidant potential (Zhang et al., 2020). The CM extracts showed strong antioxidant capacity in the TAC and FRAP experiments.The latter reconfirms our previous findings that CM extract strongly scavenges the free radicals such as DPPH, AAPH, nitric oxide, and hydrogen peroxide (Kpemissi et al., 2019b). This is further corroborated by its action in inhibiting lipoperoxidation induced by FeCl_2_-AA on organ tissues. Most organo-toxic factors, microbes, xenobiotics and others, damage the brain by inducing lipoperoxidation (Jamali-Raeufy et al., 2019). Ascorbic acid and ferrous or ferric ions are used to induce lipid peroxidation in brain tissue (Ex vivo study). As per our findings, CM extract inhibits lipoperoxidation stimulated by FeCl_2_-AA in brain tissue by significantly inhibiting the formation of MDA. This corroborated and confirmed its action in inhibiting lipoperoxidation (Kpemissi et al., 2019b). Free radicals and the damage they cause are linked to the onset, progression, and/or persistence of a number of chronic organ diseases, including neurodegenerative disorders (Onyebuchi et al., 2022). Because of the renewed importance of free radical biology and the availability of appropriate therapeutics for most of the chronic organ diseases, the role of antioxidants is well established (Mahendran et al., 2015; Wenjian et al., 2021). As a result, the exploration of innovative antioxidants in natural products, particularly plants, has received a lot of attention (Alam et al., 2006, Li and Huang, 2021). Many antioxidants derived from plants have been found to have preventive action in both primary clinical manifestations and late complications of organ chronic diseases (Haddadi et al., 2020). The phytochemical investigation of CM extract showed the presence of many distinctive bioactive constituents such as flavones, flavanones, saponins, tannins, phenolic and terpenoid compounds (Kpemissi et al., 2019b). The polyphenolic compounds in *combretum micranthum* were extracted, fractioned and isolated fractions were further puriﬁed by prep-HPLC. Further, their structures were elucidated using different spectrometric methods including UV, MS and NMR spectroscopy (Bony et al., 2014, Welch et al., 2018). Our earlier studies reconfirmed the effectiveness of flavonoids as an organo-protective compound (Kpemissi et al., 2019b, Kpemissi et al., 2020b). The search for natural medicines of plant origin with anti-inflammatory and antioxidant activities has become a point of interest because of their involvement in neuroprotection (Khokar et al., 2021). Our findings support previous findings that flavonoids have antioxidant and anti-inflammatory activities (Kpemissi et al., 2020b). Finding therapeutic agents that can counter free radicals in their oxidative and inflammatory processes such as CM extract is the highest priority in medical research today (Anadozie et al., 2018, Saleem et al., 2020, Chen et al., 2021).

The cholinergic system dysfunction might play a critical role in ND and could be the principal origin of neurocognitive problems, as well as the cognitive deficits that accompany them (Taqui et al., 2022). The most successful way and major goal for treating Alzheimer's disease is to diminish acetylcholine levels by using transitory inhibitors to block the cholinesterase that AChE and BuChE catalyze (Sang et al., 2022). Tacrine, Donepezil, Galantamine, and Rivastigmine have all been utilized as cholinesterase inhibitors (Li et al., 2016, Derabli et al., 2020). These medications are critical for the compassionate treatment of Alzheimer's disease. Nonetheless, due to their low specificity and availability, they have been linked to a number of negative impacts on some vital organs like liver (Toda et al., 2010, Farina et al., 2015). To remedy this challenge, scientists must look for a new medicine with a different mechanism of action (Cummings et al., 2022). Finding better disease-modifying medicines for ND is a critical problem for the twenty-first century (Cummings et al., 2022). According to cholinergic hypothesis, AChE inhibition has evolved as an important treatment target (Taqui et al., 2022). AChE is an enzyme involved in the cholinergic nerve system (Tang et al., 2022). Treatments aimed at reversing cholinergic deficits in ND are mostly focused on AChE inhibitors, which improve cholinergic signaling with limited and temporary therapeutic benefits (Taqui et al., 2022). Many publications have shown that cholinesterase inhibitors can be used to treat a range of conditions, including the prevention of β-amyloid plaque development, antioxidant activity, and regulation of amyloidogenic synthesis (Chauhan et al., 2022, Cummings et al., 2022). Bioactive compounds have already been shown to be potential sources of AChE inhibitors (Khokar et al., 2021). The currently licenced medications for Alzheimer's disease, galantamine and rivastigmine, are plant-derived alkaloids that provide only symptomatic relief and do not slow the course of the illness. There will be a need to look to nature for better, more effective, and lengthy drug candidates with fewer negative effects (Taqui et al., 2022). Our results show that CM can act as a potential AChE inhibitor candidate. Previous *in silico* experiments demonstrated that polyphenolic bioactive chemicals found in the CM extract inhibited Nuclear factor kappa B (NF-κB), soluble epoxide hydrolase (sEH) and activated peroxisome proliferator-activated Receptor alpha (PPARα) and gamma (PPARγ) expression (Kpemissi et al., 2019a; Kpemissi et al., 2020b). These findings are consistent with observations in the literature that flavonoids are effective neuroprotective compounds (Xie et al., 2020). These secondary metabolites from herbs seem to be a therapeutic agents in a variety of pharmacology mechanisms, including anti-inflammatory, antioxidant and neuroprotective (Welcome, 2020). In addition, blocking NF-κB and sEH as well as enabling PPARα and PPARγ expression could alleviate neurodegenerative diseases such as Alzheimer’s disease (Certo et al., 2015, Prashantha et al., 2020). All these activities of CM extract are related to neuroprotection and suggest its probable neuroprotective potential.

## 5 Conclusion

The current study demonstrated clearly that CM extract has promising antioxidant, anti-inflammatory and anticholinesterase activities. These pharmacological properties are potential targets in neuroprotection. The ex-vivo neuroprotective effects of CM extract obtained in this study could confirm its therapeutic potential in neurodegenerative disease. Furthermore*,* in order to validate the neuroprotective ability of CM extract, in vivo chronic cerebral hypoperfusion model of Alzheimer’s disease will be undertaken in our future studies.

## CRediT authorship contribution statement

**Mabozou Kpemissi:** Conceptualization, Formal analysis, Funding acquisition, Investigation, Methodology, Resources, Writing - original draft.**Yendube T. Kantati:** Conceptualization, Methodology, Data curation.**Zurina Hassan:** Funding acquisition, Data curation, Formal analysis, Supervision.**Kwashie Eklu-Gadegbeku:** Data curation, Validation, Formal analysis, Supervision.**Veeresh Prabhakar VEERAPUR:** Data curation, Supervision, Validation.
